# The impact of aqueous washing on the ability of βFeOOH to corrode iron

**DOI:** 10.1007/s11356-016-6749-3

**Published:** 2016-05-10

**Authors:** D. E. Watkinson, N. J. Emmerson

**Affiliations:** Department of Archaeology and Conservation, School of History, Archaeology and Religion, Cardiff University, John Percival Building, Colum Drive, Cardiff, CF10 3EU UK

**Keywords:** Iron, Chloride, Akaganéite, Corrosion, Treatment, Heritage, Archaeology, Management

## Abstract

Controlling the corrosion of historical and archaeological ferrous metal objects presents a significant challenge to conservators. Chloride is a major corrosion accelerator in coastal areas for historic ferrous metal structures and for the many chloride-containing archaeological objects within museums. Corrosion reactions involve the formation of akaganéite (βFeOOH) which incorporates chloride within its crystal structure and adsorbs it onto its surface. The mobility of the surface-adsorbed chloride in aqueous systems and atmospheric moisture means βFeOOH can itself cause iron to corrode. The extraction of chloride from βFeOOH by aqueous Soxhlet hot wash and aqueous room temperature washing is measured. The impact of this washing on the ability of βFeOOH to corrode iron is quantitatively investigated by determining the oxygen consumption of unwashed, Soxhlet-washed and room temperature-washed samples of βFeOOH mixed with iron powder and exposed to 80 % relative humidity. This acts as a proxy measurement for the corrosion rate of iron. The results are discussed relative to climatic factors for outdoor heritage objects and the treatment of archaeological iron in museums. Delivering better understanding of the properties of βFeOOH supports the development of evidence-based treatments and management procedures in heritage conservation.

## Introduction

### Heritage iron

The versatility of iron alloys means heritage ironwork exists as in situ structures, historical objects and archaeological artefacts excavated and stored in museums. Iron is reactive and unstable in many environments, undergoing corrosion dictated by intrinsic factors, climate and ambient environment: sulphur pollution produces sulphuric acid; coastal environments provide airborne chloride (Cl^−^); location and precipitation, temperature and wind patterns dictate the availability of moisture to support corrosion; building design and heating patterns control indoor microclimates. This study focuses on the role of the Cl^−^-bearing corrosion product akaganéite (βFeOOH) and water in the corrosion of iron in heritage and archaeological contexts.

### Chloride and corrosion of heritage ferrous metals

#### Historic objects

Atmospheric corrosion of historic iron is influenced by climatic conditions, especially relative humidity (RH), temperature, precipitation, wind speed and direction. Constant high RH can provide a continuous, thin film of moisture that is readily replenished with atmospheric oxygen to support the cathodic reaction. Large numbers of wet/dry cycles will produce extensive corrosion during drying phases that offer good oxygen permeation through the shrinking moisture film (Hœrlé et al. 2004). Electrolytes increase conductivity and hygroscopic soluble salts lower RH corrosion thresholds with their eventual deliquescence providing concentrated electrolytes (Chandler 1966). Chloride is a common contaminant of metals in the atmosphere and it forms electrolytes and hygroscopic salts. Coastal areas expose historic ferrous metals to airborne Cl^−^ deposition and archaeological objects excavated from both marine and land sites contain Cl^−^.

Marine salinity, shoreline proximity, wind direction and velocity, origin of the Cl^−^ aerosol and climatic factors such as rain events influence Cl^−^ deposition and concentration on metal surfaces (Gustafsson and Franzén 2000; Morcillo et al. 2000; Asami and Kikuchi 2003; Cole et al. 2011). Countries with long shorelines relative to land mass will offer major risk. In Cuba, Cl^−^ deposition within 250 m of the shoreline is recorded as extreme, averaging 524 mg m^−2^ day^−1^ at one site, whereas inland deposition is as low as 0.47 and 36 mg m^−2^ day^−1^ in urban areas (Corvo et al. 1995). These concentration differences are reflected in the corrosion rates of steel, with weight loss falling from 6700 g m^−2^ day^−1^ at 10 m from the shoreline to 2200 mg m^−2^ day^−1^ at 1 km inland.

The impact of rain events is complex according to the precipitation time, wetting and drying cycles and water runoff. Large amounts of rain with object morphology providing runoff will solvate and remove soluble Cl^−^, whereas high RH will influence the drying time following rain events and provide moisture films that solvate Cl^−^ and deliver electrolyte. The form of Cl^−^ salt will dictate hygroscopicity and may facilitate rusting at low humidity (Evans and Taylor 1974). Geography may be overruled by context as mild steel in an inland, rural area of Japan produced Cl^−^-containing βFeOOH as a corrosion product over 17 years due to road salting (Asami and Kikuchi 2003). Historical and archaeological objects may retain Cl^−^ from their use life and burial contexts.

Atmospheric corrosion of iron produces predominantly goethite (αFeOOH), lepidocrocite (γFeOOH) and magnetite (Fe_3_O_4_) (Hœrlé et al. 2004; Asami and Kikuchi 2003; Morcillo et al. 2011). Rain events produce a wetting phase where anodic dissolution of iron is balanced by the reduction of ferric species to less oxidised phases, followed by a wet phase involving cathodic reduction of oxygen and, finally, oxidation of ferrous ions and the reduced species from initial wetting occurs during a drying phase (Dillmann et al. 2004; Hœrlé et al. 2004; Stratmann and Streckel 1990). Sufficiently high Cl^−^ concentration (Rémazeilles and Refait 2007) and favourable environmental factors produce βFeOOH as a corrosion product. Its presence has been strongly linked with Cl^−^ contamination of iron in coastal areas (Maeda et al. 1992; Yamashita et al. 2001; Asami and Kikuchi 2003) and object provenance influences its occurrence, such as on the 1843 wrought iron hull of Brunel’s *ss* Great Britain housed in a dry dock in Bristol after a long life at sea (Watkinson and Lewis 2004).

#### Archaeological objects

During burial in moist environments, archaeological iron attracts highly soluble mobile Cl^−^ ions to its metal surface to counter the charge buildup of Fe^2+^ at anode sites, producing a solution of FeCl_2_ at the metal surface below corrosion product layers (Turgoose 1982a). Chloride has also been detected as ferrous hydroxychloride (βFe_2_(OH)_3_Cl) located close to the metal surface on excavated objects that have not been allowed to undergo post-excavation oxidation (Réguer et al. 2007). These soluble compounds offer a supply of Cl^−^ to support corrosion and are noted to be a precursor to the formation of βFeOOH in the oxidative post-excavation atmosphere. Corrosion product layers on archaeological iron comprise mostly goethite (αFeOOH), magnetite (Fe_3_O_4_), maghemite (γFe_2_O_3_) and sometimes siderite (FeCO_3_) with silica (SiO_2_) inclusions from the soil (Neff et al. 2007). Ingress of oxygen through these often dense layers is limited, and this facilitates the survival of metal cores over long burial periods (Neff et al. 2005). During burial, iron objects accumulate significant amounts of Cl^−^ related to site geography, soil properties, burial context, precipitation and intrinsic properties such as slag content and work history. Chloride concentrations of 500–2500 ppm are common for terrestrial iron (Watkinson 2010) and iron from marine contexts may reach 50,000 ppm.

Post-excavation, damp iron objects dry out and Fe^2+^ in the FeCl_2_ solution next to their metal surface hydrolyses, producing a low pH (Turgoose 1982b). Abundance of Cl^−^ and Fe^2+^ coupled with low pH favour the formation of the iron hydroxyoxide βFeOOH polymorph. It adsorbs Cl^−^ onto its surface and incorporates it into tunnels within its crystal structure (Ståhl et al. 2003), with its formation relating to the [Cl^−^]/[Fe^2+^] ratio in solution. In FeCl_2_/NaOH solutions, it does not form below 1.6 M Cl^−^ but it is the sole product above 3.2 M, whereas high [Cl^−^] but low [Fe^2+^] produces only α- and γFeOOH (Rémazeilles and Refait 2007). Intermediates such as βFe_2_(OH)_3_Cl and green rusts precede its formation, which is likely why low [Fe^2+^] prevents its occurrence since in these conditions the necessary intermediates do not form.

The chloride content of βFeOOH varies. Reported concentrations range from 1.3 to 17 %, *w*/*w* (Childs et al. 1980; Ishikawa and Inouye 1975; Keller 1970; Watkinson and Lewis 2005a) with βFeOOH on archaeological objects post-excavation returning 3–14.8 % (*w*/*w*) Cl^−^ (Thickett and Odlyha 2014). Differences in Cl^−^ content likely result from Cl^−^ and Fe^2+^ concentration differences and the synthesis method, such as acid precipitation from solution (Atkinson et al. 1977; Rémazeilles and Refait 2007) or corrosion of iron in high chloride concentrations and high RH (Turgoose 1982b).

### βFeOOH as a corrosion threat to iron

The occurrence of βFeOOH is symptomatic of post-excavation corrosion of chloride-infested archaeological iron (Zucci et al. 1977) which can occur after decades within museum stores and displays due to raised RH and the residual Cl^−^ within objects. βFeOOH is hygroscopic (Kaneko and Inouye 1979; Watkinson and Lewis 2005b) and corrodes iron that is in contact with it. Watkinson and Lewis (2004) detected this occurring at 16 % RH, but not at 12 % RH, whilst Thickett and Odlyha (2014) detected corrosion as low as 13 % RH. The accuracy and tolerance of equipment used to control RH at low values means that the exact cutoff RH for corrosion is difficult to identify, but reaction at low RH is slow and only begins to accelerate appreciably around 40 % RH (Watkinson and Lewis 2004). The corrosion rates for electrolyte-driven reactions increase significantly around 55–60 % RH, where the adsorbed water films are two to five monolayers thick and clustered, increasing to continuous films of six to ten monolayers at 80 % RH (Leygraf and Graedel 2000, pp. 11, 283; de Rooij 1989). Capillaries of 1.5 nm will condense water at 50 % RH in thick, porous corrosion product layers, lowering the corrosion threshold humidity (Garverick 1994). Besides supporting electrochemical corrosion down to low humidity levels, βFeOOH is physically dangerous; its tower-like crystal growth causes overlying corrosion layers to crack and exfoliate. Whilst the mobile surface-adsorbed Cl^−^ on βFeOOH is removed by washing (Watkinson and Lewis 2005b; North 1982), Cl^−^ locked within the crystal structure remains but poses no corrosion threat (Réguer et al. 2009). Additionally, removing soluble chloride reduces the hygroscopicity of βFeOOH (Watkinson and Lewis 2005b) which prevents it corroding iron at low humidity since it cannot attract water to mobilise Cl^−^.

### Preventing chloride-driven corrosion of iron

Only desiccation (Watkinson and Lewis 2005a) or deoxygenation can prevent the corrosion of heritage iron entirely. Treatments aiming to reduce corrosion rates have been developed, the most extensively researched being aqueous alkaline washing that aims to extract soluble Cl^−^ by solvation of solid FeCl_2_ present on dry iron and mobile Cl^−^ on βFeOOH (Watkinson et al. 2013). The outcomes of these treatments are unpredictable and current studies suggest the iron is rarely stabilised, but they do reduce the corrosion rate (Rimmer et al. 2013).

For Cl^−^-contaminated historical iron exposed outdoors, coatings are often employed to protect against corrosion. Should these be absent, inexpertly applied or lacking maintenance, corrosion will ensue from water supplied by high RH and precipitation. The influence of rainfall on corrosion is complex; more rain may be beneficial in reducing the Cl^−^ content by removing surface-adsorbed Cl^−^ from βFeOOH and solvating FeCl_2_, but the pattern of rain is important as frequent short showers will induce many wetting/drying cycles, offering greater opportunity for corrosion when compared to continuous rain that flushes out chloride with fewer wetting/drying cycles.

The research reported here was designed with these factors in mind and with an appreciation of the importance for heritage conservation of understanding the impact of washing βFeOOH on the corrosion of iron. It will contribute to elucidating treatments for chloride removal from archaeological iron and for creating storage guidelines, as well as designing and managing protective measures for heritage iron exposed to the atmosphere.

### Aim and objectives

#### Aim

- Determine the impact of washing βFeOOH on its ability to corrode iron in high relative humidity.

#### Objectives

- Synthesise βFeOOH and quantitatively determine its rate of corrosion of iron.
- Wash βFeOOH in water until no more Cl^−^ is removed.
- Quantitatively determine βFeOOH post-washing rate of corrosion of iron.

## Method

### Akaganéite preparation

βFeOOH was synthesised via a solid-state reaction between pure iron and ferrous chloride. One hundred grams of pure iron powder (Sigma-Aldrich) and 100 g ferrous chloride tetrahydrate (FeCl_2_⋅4H_2_O) (Sigma-Aldrich) were mixed and exposed for 4 months in open Petri dishes within a humidification chamber in which a saturated solution of sodium carbonate (Na_2_CO_3_) produced 92 % ± 5 RH. Oxygen in the chamber was replenished regularly. The corrosion product was washed in acetone using a Buchner funnel until the wash was colourless to remove excess ferrous chloride. Success of the synthesis and washing was determined by X-ray diffraction (XRD) assay of the resultant powder using a PANalytical X'Pert Pro (CuΚ_α_) X-ray powder diffractometer. βFeOOH (0.1 g) was digested in 5 M nitric acid (AnaLar Grade) and ferrous ions in the digestion solution were precipitated by the addition of excess 3 M NaOH and filtered out using Whatman no. 1 filter paper. The filtrate was rinsed with deionised water into the digestion solution and the pH measured using an Electronic Instruments 7065 pH/mV meter and adjusted to pH 6.6 by the addition of HNO_3_. The solution was then buffered and the chloride content measured as described below.

### Hot and room temperature washing of βFeOOH

βFeOOH (2.5 g) was hot washed using a Soxhlet extraction system comprising a reservoir flask containing 400 ml deionised water and anti-bumping granules, a 100 ml Soxhlet and a condenser. The βFeOOH was contained within a Whatman cellulose extraction thimble (internal dimension, 28 × 80 mm) to retain it within the Soxhlet. The Soxhlet refilled every 20 min with deionised water at 76 °C. The wash solution was sampled at intervals by the extraction of 10 ml samples from the reservoir flask with the Soxhlet fully emptied. The chloride concentration in the samples was measured according to the protocol outlined below and the wash halted once the Cl^−^ concentration in the wash stabilised.

A further 2.0 g βFeOOH was washed in a Whatman cellulose extraction thimble (described above) suspended in a covered beaker containing 400 ml deionised water at room temperature. The thimble avoided loss of βFeOOH during sampling from the solution. The extraction solution was stirred throughout the wash. Of the wash solution, 10 ml samples were extracted at intervals and the Cl^−^ concentration measured. The wash was stopped once the chloride concentration in the wash solution stabilised. Post-washing, the hot- and room temperature-washed samples were dried within their thimbles in a closed polypropylene container with desiccated silica gel to create a low RH environment.

### Chloride concentration measurement

The chloride concentration of the samples was measured using a Radiometer Analytical PHM250 specific ion meter with a mercury/mercury sulphate reference electrode (REF621) and chloride-specific electrode (ISE25Cl) capable of detecting Cl^−^ down to concentrations of approximately 1 ppm. The 10 ml samples were buffered with 1 ml 0.5 M ammonium acetate/0.5 M acetic acid solution to standardise ionic activity before measurement and the meter calibrated every 4 h using four standard solutions made up from a 0.1 M (3544 ppm) NaCl aliquot prepared from an ampoule.

### Post-wash rinsing

Samples (0.1 g) of the hot- and room temperature-washed βFeOOH were rinsed with 10 ml deionised water in a funnel through Whatman no. 1 filter paper. The rinses were repeated until the Cl^−^ concentration (measured as above) in the filtrate was approximately 6 ppm. The total weight per cent Cl^−^ rinsed from each sample was calculated.

### Examining the effect of containment within a thimble on chloride release

The impact on the rate of Cl^−^ release of containing the βFeOOH in a thimble during washing was examined by room temperature washing of two samples of 0.5 g βFeOOH, one in a thimble and one as a free powder, both in 200 ml deionised water in covered beakers with continuous stirring. Five millilitre samples of the wash solutions were extracted by pipette at regular intervals and the Cl^−^ concentration measured according to the protocol above. Stirring was stopped in the free powder sample wash solution and the βFeOOH allowed to settle before the samples were taken. The samples were filtered to remove the negligible amounts of βFeOOH particulate before Cl^−^ measurement.

### Continuous chloride release in the initial wash period

The rate of Cl^−^ release over the first 20 min of room temperature washing was examined by continuous Cl^−^ measurement in a room temperature wash of βFeOOH. Of the βFeOOH, 0.25 g was washed as a free powder (not contained in a thimble) in 100 ml of continuously stirred deionised water. The wash solution was buffered with 10 ml 0.5 M ammonium acetate/0.5 M acetic acid solution and the Cl^−^ measured using the PHM250 specific ion meter. Measurements were made soon after the βFeOOH entered the deionised water and as frequently thereafter as the operation of the meter permitted.

### X-Ray diffraction

X-Ray diffraction of the unwashed and hot- and room temperature-washed βFeOOH was carried out using a PANalytical X'Pert Pro (CuΚ_α_) X-ray powder diffractometer to identify any changes in the composition of the powders following washing. XRD data were processed using PANalytical X’Pert HighScore software.

### Corrosion rate determination by oxygen consumption

Assuming that the predominant reaction in the corrosion of iron powder mixed with βFeOOH is the conversion of Fe to FeOOH, oxygen consumption during a reaction can be used as a proxy corrosion rate measurement. Samples were prepared by grinding 0.05 g βFeOOH (dried in a low RH environment) with 0.05 g Fe powder in an agate mortar and pestle, placed in individual weigh boats and each sealed within a reaction vessel (250 ml Mason Ball glass jars with plastic-coated brass sealing discs tightened with threaded outer sealing rings of brass, which create a seal by deforming a synthetic rubberised ring on the disc) containing 165 g silica gel conditioned to 80 % RH. Within each vessel was an oxygen sensor spot (World Precision Instruments (WPI) part #503090) adhered to the interior wall of the vessel using silicon adhesive (Radio Spares RTV silicone rubber compound). The reaction vessels with samples were stored in a Binder KB720 climate chamber to control the temperature to 20 ± 0.5 °C and avoid RH changes within the vessels which would result from fluctuating temperature. Madgetech RHTemp 101A data loggers monitored the internal environment. Using a fibre-optic probe and WPI OxyMini oxygen meter (WPI OXY-MINI-AOT with cable #501644), the oxygen concentration within each vessel was measured at 5 min intervals throughout the test period.

The accuracy of the oxygen measurements is ±2 mbar at an atmospheric oxygen pressure of 210 mbar and increases proportionally with decreasing oxygen pressure. Control vessels filled with nitrogen showed a negligible ingress of oxygen over a 2 year period, indicating very little leakage of the vessels (Watkinson and Rimmer 2014). Consumption of the reaction vessel, silica gel, weigh boat and data logger was recorded and found to be negligible.

The oxygen consumption of two samples of unwashed, hot-washed and room temperature-washed βFeOOH with Fe powder at 80 % RH was recorded. One sample of the rinsed hot-washed and one rinsed room temperature-washed βFeOOH with Fe powder were also recorded. The oxygen consumption of 0.05 g of unmixed unwashed, hot-washed and room temperature-washed βFeOOH and 0.05 g Fe powder was also recorded to eliminate oxygen consumption of the individual powders as the cause of oxygen consumption in the corrosion tests of βFeOOH with Fe powder.

## Results

### XRD assay of βFeOOH as synthesised

**Fig. 1:**
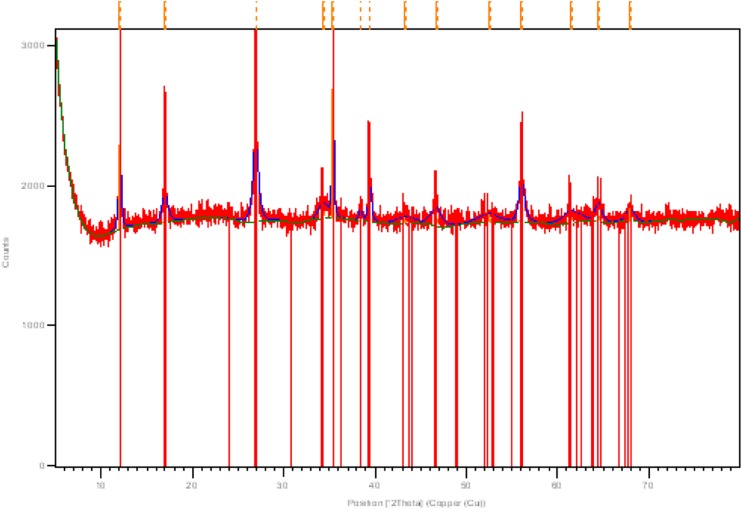
XRD pattern of unwashed βFeOOH (diffraction code 00-042-1315)

The XRD of the unwashed βFeOOH determined its composition to be 100 % akaganéite (diffraction code 00-042-1315). The diffraction pattern is given in Fig. 1. The digestion of βFeOOH returned 7.8 % (*w*/*w*) Cl^−^ (Table 1). This is within the expected *% w/w* range of Cl^−^ contained in βFeOOH (Childs et al. 1980; Ishikawa and Inouye 1975; Keller 1970; Watkinson and Lewis 2005a; Thickett and Odlyha 2014).

**Table 1:** Values (% *w/w*) for chloride extracted and residual in unwashed, washed and rinsed samples of βFeOOH

βFeOOH sample	Cl^−^ removed during washing (% *w*/*w*)	Cl^−^removed during rinsing (% *w*/*w*)	Total Cl^−^ removed (% *w*/*w*)	Cl^−^ remaining (% *w*/*w*)
Unwashed	–	–	–	7.8
Soxhlet hot washed	5.5	–	5.5	2.3
Soxhlet hot washed and rinsed	5.5	0.4	5.8	2.0
Room temperature washed	3.9	–	3.9	3.9
Room temperature washed and rinsed	3.9	1.3	5.2	2.6

### βFeOOH Soxhlet hot and room-temperature washing in thimble

**Fig. 2:**
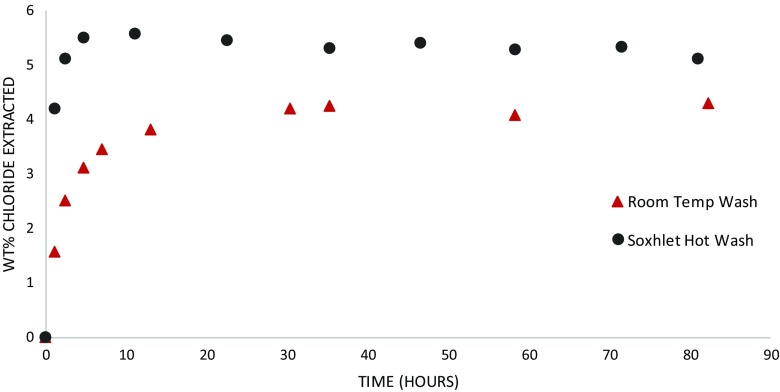
Weight of chloride in hot wash and room temperature wash solutions during wash period as a percentage of total mass βFeOOH

Both Soxhlet hot washing and room temperature washing of βFeOOH released Cl^−^ into the wash solution. Figure 2 shows the *% w/w* Cl^−^ from βFeOOH in the solutions as washing progressed. Table 1 records the total Cl^−^ removed. Hot washing released Cl^−^ at a faster initial rate than did room temperature washing, and the amount of Cl^−^ in the wash solution stabilised earlier and at a higher value than that with room temperature washing. Soxhlet hot wash released 5.5 % (*w*/*w*) Cl^−^ at the 80 h plateau, and room temperature washing over the same time period released 3.9 % (*w*/*w*) Cl^−^ (Table 1 and Fig. 2).

In all the tests, the release of Cl^−^ into deionised water produced a pH of 2.5 ± 0.2. Whilst all βFeOOH in the stirred room temperature wash tests continuously remained at this pH, whether as a free powder or contained within a cellulose thimble, in contrast, βFeOOH within the Soxhlet hot wash was repeatedly immersed in deionised water.

### Post-wash rinsing

Rinsing both hot- and room temperature-washed βFeOOH with deionised water resulted in further release of Cl^−^ (Table 1). A concentration of approximately 6 ppm Cl^−^ in the rinse solution was achieved by rinsing with 35 ml deionised water for the Soxhlet hot-washed βFeOOH and 70 ml deionised water for the room temperature-washed βFeOOH. Rinsing removed a further 0.4 % (*w*/*w*) Cl^−^ from the Soxhlet hot-washed βFeOOH and 1.3 % (*w*/*w*) Cl^−^ from the room temperature-washed βFeOOH (Table 1).

### Chloride release from thimble and free powder

**Fig. 3:**
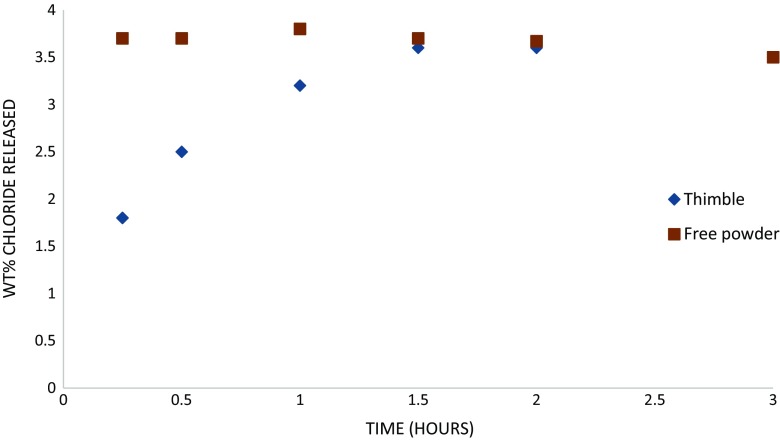
Chloride release (% *w/w*) over 3 h of room temperature washing of βFeOOH contained in a thimble and as a free powder

The measurements of Cl^−^ extraction from equivalent masses of βFeOOH contained in a thimble and as a free powder during washing show that the initial rate of release of Cl^−^ into the wash solution is reduced by containment as compared to the free powder (Fig. 3). Total Cl^−^ extracted over 3 h was the same in both cases, with broadly equivalent weight per cent of Cl^−^ extracted by 1.5 h of washing.

### Continuous chloride release in the initial wash period

**Fig. 4:**
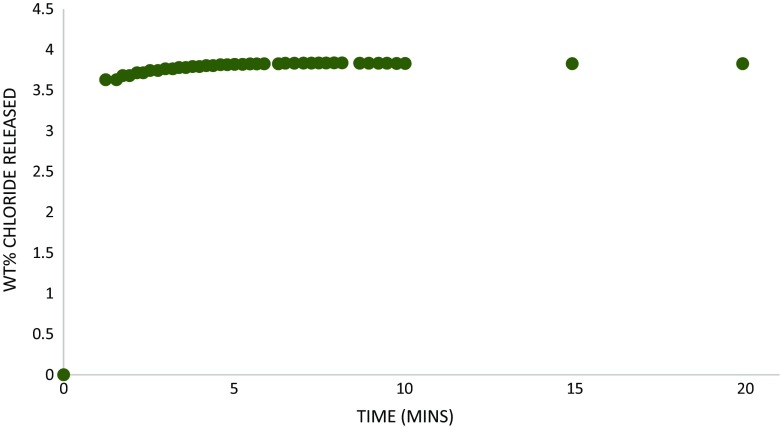
Initial chloride release (% *w/w*) from room temperature washing of βFeOOH as a free powder

Continuous measurement of Cl^−^ extraction from a free powder in stirred deionised water at room temperature over an initial 20 min wash period demonstrated that the majority of the total Cl^−^ release occurred within the first minute of washing (Fig. 4). Of the Cl^−^ extracted during this test, 95 % had been released by the first measurement at 74 s. The total Cl^−^ extracted over 20 min matched the total Cl^−^ extracted over 180 min in the longer free powder room temperature wash (Fig. 3). This indicated that the 0.5 M acetic acid/ammonium acetate buffer added to the wash solution (1:10 ratio) to allow continuous accurate reading of Cl^−^ using the specific ion electrodes did not impact on either the Cl^−^ extraction rate or the total amount of chloride extracted in the wash.

### XRD assay of hot- and room temperature-washed βFeOOH

**Fig. 5:**
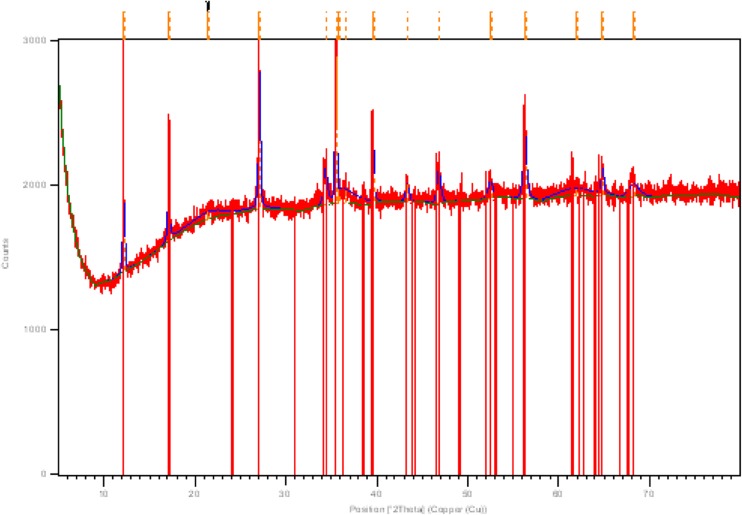
XRD pattern of Soxhlet hot-washed βFeOOH (diffraction code 00-042-1315)

**Fig. 6:**
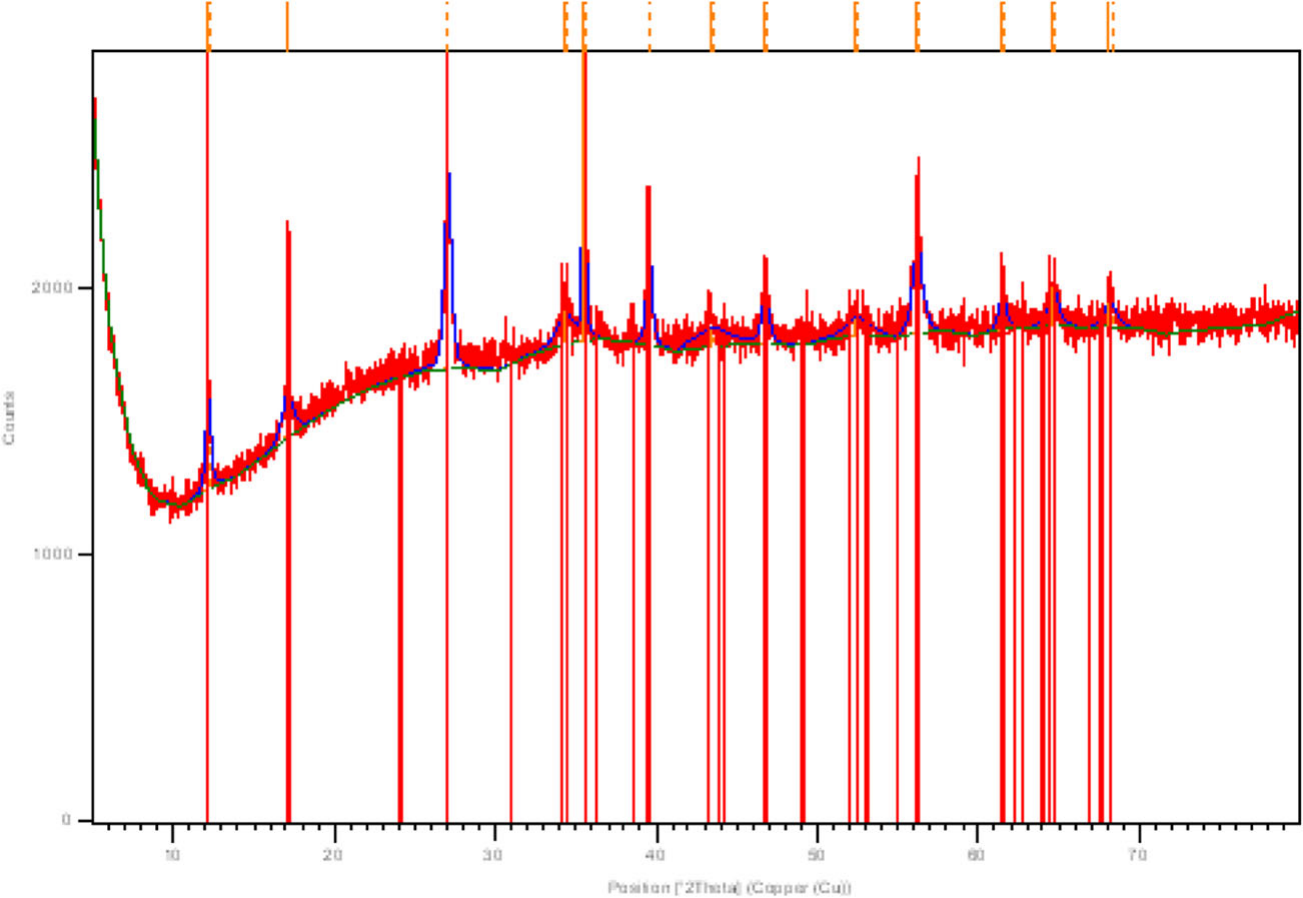
XRD pattern of room temperature-washed βFeOOH (diffraction code 00-042-1315)

The XRD of the hot- and room temperature-washed βFeOOH samples determined the composition to be 100 % akaganéite (diffraction code 00-042-1315), indicating that no transformation resulted from either washing procedure (Figs. 5 and 6).

### Oxygen consumption and corrosion

None of the unmixed unwashed and washed βFeOOH or iron powder controls the consumed oxygen at 80 % RH over a period of 20 h. Any oxygen consumption of the mixed βFeOOH/Fe powder samples can therefore be attributed to the corrosion of the iron.

**Fig. 7:**
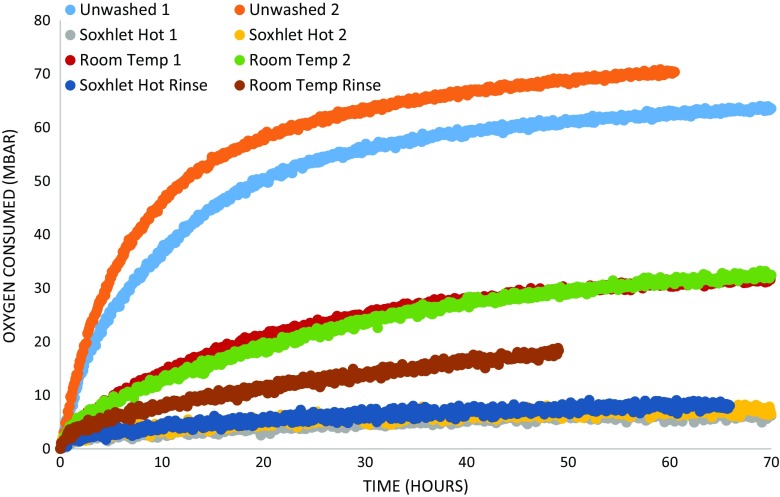
Oxygen consumption of 0.05 g washed, unwashed and rinsed βFeOOH samples mixed with 0.05 g iron powder at 80 % RH

**Fig. 8:**
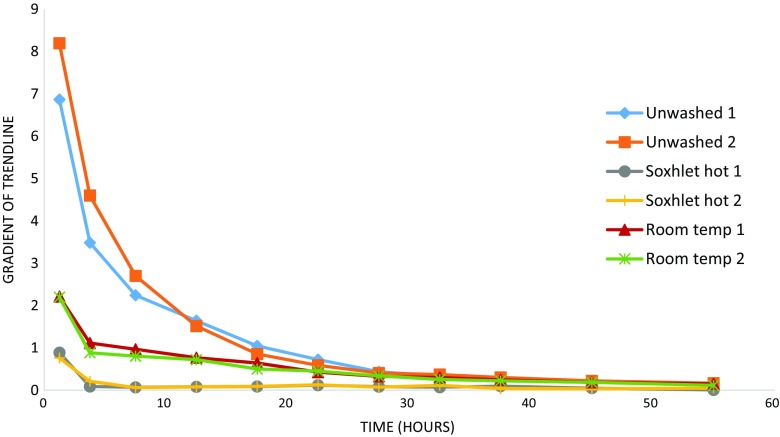
Oxygen consumption rates as gradients. Data points plotted at midpoint of gradient time period

All samples of βFeOOH mixed with iron powder consumed oxygen at 80 % RH over the test period, so can be said to have corroded (Fig. 7). An initial faster corrosion rate is recorded for all samples which reduces over time, as shown by the drop-off of the gradient of the trend line in all cases (Fig. 8). The initial period of rapid oxygen consumption is likely due to the finely powdered iron offering a large surface area for corrosion aided by its intimate mixing with the βFeOOH. Slowing occurs with the depletion of iron by corrosion and the formation of corrosion products around the iron particles hindering the corrosion process. Upon completion of the tests, the unwashed βFeOOH/iron mixtures had become more compacted.

None of the reactions goes to completion during the test period, but all reduce to a similar slow rate. The initial corrosion rate and total corrosion are greatest for the unwashed βFeOOH samples. The corrosion rate and total corrosion are reduced for the room temperature-washed βFeOOH in comparison to the unwashed samples and reduced still further for the Soxhlet hot-washed samples. Rinsing the room temperature-washed βFeOOH is seen to reduce corrosion, but this remains greater than for the Soxhlet hot-washed samples. Rinsing the Soxhlet hot-washed sample does not reduce its oxygen consumption rate below the unrinsed sample. This is reflected in the very small amount of extra Cl^−^ removed in the rinse process (Table 1). There is good agreement between the corrosion rates for the samples washed in the same manner, demonstrating reproducibility in the oxygen consumption measurement method.

## Discussion

### Removal of chloride from βFeOOH

**Fig. 9:**
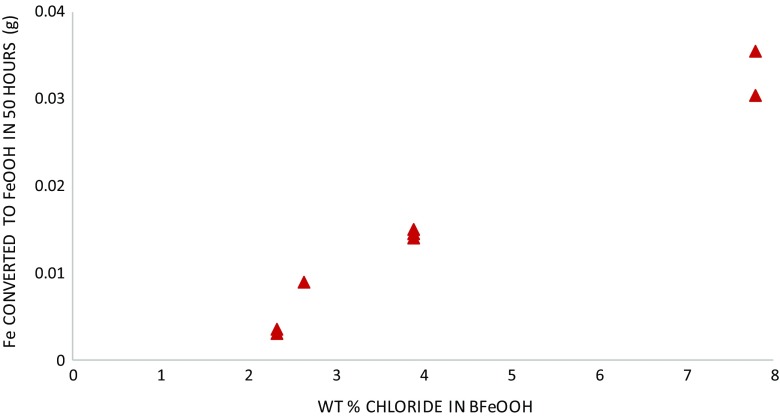
Graph showing the correlation between the weight per cent chloride in βFeOOH samples and the mass of Fe powder converted to FeOOH when mixed with these βFeOOH samples at 80 % RH after 50 h. This is calculated from the oxygen consumed during the reactions using the ideal gas law and assumes that conversion to FeOOH is the only reaction occurring (Emmerson and Watkinson 2014, 2016)

Soxhlet hot washing removes more chloride than did room temperature washing (Fig. 2). Calculation reveals that residual Cl^−^ in βFeOOH for the Soxhlet hot-washed sample is 2.0 % (*w*/*w*) and for the room temperature-washed βFeOOH is 2.5 %, *w*/*w* (Table 1). The amount of Cl^−^ remaining matches typical levels of Cl^−^ held within the crystal structure of βFeOOH (North 1982), indicating that most of the mobile surface-adsorbed Cl^−^ has been removed. This explains the reduced oxygen consumption rate of iron that results from washing and the difference in oxygen consumption between the Soxhlet- and room temperature-washed samples and their rinsed counterparts (Figs. 7 and 8 and Table 1). The extent to which βFeOOH is able to corrode iron reflects the amount of chloride removed by washing; the more chloride that is removed, the slower the oxygen consumption and the less the oxidation of iron (Fig. 9). This is not an unexpected relationship. The quantity of mobile chloride available on the βFeOOH will be an important factor for determining the corrosion rate of iron, likely due to its ability to act as an electrolyte and its influence on the hygroscopicity of βFeOOH (Watkinson and Lewis 2005b). Comparing the two washing methods, either or both the elevated temperature of Soxhlet washing or its continual renewal of ion-free wash solution lead to improved removal of Cl^−^. The use of a thimble to contain the βFeOOH slows the initial release of Cl^−^ as compared to free powder, but it does not alter the end point of the wash method (Fig. 3).

### Context for historical and archaeological ferrous metals

#### Outdoor heritage structures

The impact of climate on historical ferrous metals containing βFeOOH as a corrosion product can be beneficial. The release of large amounts of Cl^−^ from βFeOOH, within minutes of its immersion in water at room temperature, indicates that rain events can be expected to remove most of the mobile Cl^−^ from βFeOOH (Figs. 3 and 4), provided there is a runoff route for the rain solvating the Cl^−^. The oxygen consumption tests reveal that this significantly reduces its aggressiveness towards iron (Figs. 7 and 9) and additional washing with ion-free water further reduces Cl^−^ and the ability of βFeOOH to corrode iron (Table 1 and Fig. 7). Prolonged rain will not be necessary to remove all the surface-adsorbed Cl^−^ it is possible to remove at ambient temperature. The likely positioning of βFeOOH within the rust layers of iron alloys corroding in the atmosphere will make washing off its mobile Cl^−^ easier. Analysis of cross-sections of rust on carbon steel exposed for 17 years under a bridge in a coastal area of Japan pinpointed βFeOOH as occurring at the surface of the rust layer and extending 40 % within its overall thickness (Asami and Kikuchi 2003). Its location will offer better opportunity to wash off adsorbed Cl^−^ compared to positioning of βFeOOH next to the metal surface, which is normally the situation for archaeological iron that has undergone post-excavation corrosion (Neff et al. 2005).

The effectiveness of rain at removing Cl^−^ will be influenced by object geometry. The Cl^−^ solution formed by the rain requires a runoff route; otherwise, the combination of high electrolyte concentration during the wet phase and concentrating Cl^−^ in solution as evaporation progresses during the drying phase will support corrosion. Ambient RH will affect the drying rate and the occurrence and longevity of partially saturated rust pores during the wet–dry phase (Dillmann et al. 2004) which will not allow complete runoff of the Cl^−^-containing rainwater. The Cl^−^ retained will contribute to corrosion. Additionally, a low pH from the hydrolysis of anodically produced Fe^2+^ ions would likely lead to the formation of fresh βFeOOH in the presence of sufficient Cl^−^. The absence of a runoff route for the rain will also prevent the occurrence of a concentration gradient that would diffuse solvated Cl^−^ away from the βFeOOH, allowing it to readsorb during the drying phase. Diffusion will impact on the removal of Cl^−^, but it may not be that significant as although diffusion of βFeOOH washed at room temperature in the thimble was slower than the free powder wash at the same temperature, it soon reached the same end point (Fig. 3).

Since the results indicate that temperature likely influences the amount of mobile Cl^−^ removed from βFeOOH (Fig. 2 and Table 1), warm rain might be expected to remove more Cl^−^ than its cold equivalent. More precise investigation over a range of temperatures representative of climatic zones would clarify this. The oxygen consumption tests reported here indicate that high RH climates will activate βFeOOH by mobilising its surface-adsorbed Cl^−^ and rain events cannot be expected to stop this entirely, as evidenced by continued but slower corrosion of iron by the βFeOOH washed at room temperature (Figs. 7 and 8). Overall, climate and precipitation influence corrosion in many ways, and the contribution of βFeOOH to the corrosion rate of ferrous metals, via the routes identified in this paper, may be negligible in comparison to other variables.

#### Archaeological iron

The results here indicate that washing methods designed to remove soluble Cl^−^ from iron will benefit from the use of elevated temperature. Many such treatments have been employed (Knight 1997). The better Cl^−^ extraction efficiency from the βFeOOH at higher temperatures means its future contribution to continued corrosion of the iron will be negligible. Hindrance to effective washing of βFeOOH arises from its location on archaeological iron as it is normally next to the metal surface under dense corrosion product layers millimetres thick. Accessing this βFeOOH to solvate its adsorbed Cl^−^ and diffuse it out of the object into the wash solution will be difficult. This contrasts with ferrous metals in the atmosphere where βFeOOH predominates in the upper outer surface of rust. Washing archaeological iron often employs alkaline solutions, and these will flood the βFeOOH surface with OH^−^ ions, offering opportunity for ion exchange with the Cl^−^ (Watkinson et al. 2013). Elevated temperature could still be expected to benefit the washing process, and experimental study could verify this.

The corrosion tests run in this study provide worst-case scenarios. Iron powder presents a large surface area for reaction, and the βFeOOH is intimately mixed with it by grinding the two together. In a normal corrosion context, the βFeOOH is likely to be localised on any object by the occurrence of low pH and high Cl^−^ concentration necessary for it to form. Equally, it will only be in contact with a small area of ferrous metal, which will likely support pitting corrosion, and this may make the βFeOOH even less accessible. The corrosion rates identified in these tests cannot be extrapolated in any quantitative form, but they do show rapid initial corrosion followed by slowing. Similarly, it is shown quantitatively that washing reduces the ability of βFeOOH to corrode iron.

## Conclusion

The chloride content of synthetic βFeOOH can be significantly reduced within minutes in aqueous wash systems. A plateau for Cl^−^ extraction is reached in a few hours in hot wash and within a couple of days in a room temperature wash. Both washing methods reduce the ability of βFeOOH to corrode iron at high humidity, but neither stops corrosion entirely, which is shown to be proportional to the amount of Cl^−^ removed. This data indicates that for outdoor heritage iron with βFeOOH as a corrosion product, rain events will be beneficial for reducing corrosion caused by adsorbed water-soluble Cl^−^ on βFeOOH. Washing archaeological iron to solvate Cl^−^ attracted during burial will significantly reduce the ability of βFeOOH to corrode it, with heated solutions offering the biggest reduction. Provided there is unimpeded access to the βFeOOH, washing need only last a few days to remove all extractable Cl^−^. This means the continuity, density and integrity of corrosion product layers, as determinants of object porosity, will influence access to the underlying βFeOOH and outward diffusion of its chloride.

Better understanding of the mechanisms occurring during washing of archaeological iron, provided by the data from this experimental study, allows development of evidence-based washing methods in conservation practice. This benefits management practices for the storage and display of archaeological objects. Similarly, management of outdoor heritage is aided by knowledge of the role of βFeOOH in the corrosion of iron and the impact of climate on this role.
